# The Warburg effect in human pancreatic cancer cells triggers cachexia in athymic mice carrying the cancer cells

**DOI:** 10.1186/s12885-018-4271-3

**Published:** 2018-04-02

**Authors:** Feng Wang, Hongyi Liu, Lijuan Hu, Yunfei Liu, Yijie Duan, Rui Cui, Wencong Tian

**Affiliations:** 1 The Institute of Integrative Medicine for Acute Abdominal Diseases, Nankai Hospital, No. 6, Changjiang Road, Nankai, Tianjin, 300100 China; 2 The Post-doctoral Working Station, Tianjin Medical University, Tianjin, 300070 China; 3 Present Address: The Centre of Disease Control, Dagang, Tianjin, 300270 China

**Keywords:** Cancer cachexia, The Warburg effect, Pancreatic cancer, Cytokines, Mouse

## Abstract

**Background:**

Cancer cachexia is a cancer-induced metabolic disorder and a major cause of cancer-induced death. The constituents of cancer cachexia include an increase in energy expenditure, hepatic gluconeogenesis, fat lipolysis, and skeletal-muscle proteolysis and a decrease in body weight. The aetiology of cancer cachexia is unclear and may involve cancer-cell metabolism and secretion. In this study, we investigated whether the high glycolysis in cancer cells (the Warburg effect) triggers cachexia in athymic mice carrying pancreatic cancer cells.

**Methods:**

First, we examined five human pancreatic cancer cell lines for glycolysis and cachectic-cytokine secretion. Consequently, MiaPaCa2 and AsPC1 cells were selected for the present study, because the glycolysis in MiaPaCa2 cells was typically high and that in AsPC1 cells was exceptionally low. In addition, both MiaPaCa2 and AsPC1 cells were competent in the secretion of examined cytokines. Next, we transplanted MiaPaCa2 and AsPC1 cells subcutaneously in different athymic mice for 8 weeks, using intact athymic mice for control. In another experiment, we treated normal mice with the supernatants of MiaPaCa2 or AsPC1 cells for 7 days, using vehicle-treated mice for control. In both models, we measured food intake and body weight, assayed plasma glucose, triglycerides, and TNF-α and used Western blot to determine the proteins that regulated hepatic gluconeogenesis, fat lipolysis, and skeletal-muscle proteolysis in the corresponding tissues. We also studied the effect of MiaPaCa2-cell supernatants on the proteolysis of C2C12 skeletal-muscle cells in vitro.

**Results:**

The athymic mice carrying high-glycolytic MiaPaCa2 cells had anorexia and also showed evidence for cachexia, including increased hepatic gluconeogenesis, fat lipolysis and skeletal-muscle proteolysis and decreased body weight. The athymic mice carrying low-glycolytic AsPC1 cells had anorexia but did not show the above-mentioned evidence for cachexia. When normal mice were treated with the supernatants of MiaPaCa2 or AsPC1 cells, their energy homeostasis was largely normal. Thus, the cachexia in the athymic mice carrying MiaPaCa2 cells may not result from humeral factors released by the cancer cells. In vitro*,* MiaPaCa2-cell supernatants did not induce proteolysis in C2C12 cells*.*

**Conclusion:**

The Warburg effect in pancreatic cancer cells is an independent aetiological factor for pancreatic cancer-induced cachexia.

## Background

Cancer cachexia is a metabolic syndrome present in 50% of all cancer patients and more frequent in the patients with pancreatic cancer [1–4]. The components of cancer cachexia include increased energy expenditure, augmented hepatic gluconeogenesis, uncontrolled fat lipolysis, unrestrained skeletal-muscle proteolysis, and decreased body weight [1, 2]. How these pathologies are initiated to induce cachexia is unclear, but several hypotheses are proposed. For instance, cachectic cytokines such as tumour necrosis factor-α (TNF-α), interferon-γ (IFN-γ), and different interleukins (ILs., e.g. IL-1β and IL-6) may be increased in the peripheral circulation of cancer patients and induce cancer cachexia [1, 2, 5]. In addition to regular cytokines, cancer cells may release cachectic proteins that are not available in normal subjects, such as lipid mobilizing factor (LMF) and proteolysis inducing factor (PIF) [6–9]. Further, the endocrine pancreas may be impaired in cancer patients and, thereafter, involved in cancer cachexia [3, 4, 10–12]. Last but not least, cancer cachexia may be triggered by cancer-cell glycolysis [13–17].

Mammalian cells produce energy primarily by oxidative phosphorylation (36 ATP/glucose). However, cancer cells switch their major way of energy production from oxidative phosphorylation to glycolysis (2 ATP/glucose). The aberrant way of energy production in cancer cells is known as the Warburg effect [18]. To get enough energy by glycolysis, cancer cells over-express key regulators of glycolysis, such as glucose transporters and glycolytic enzymes. Cancer-induced hypoxia-inducible factor-1α (HIF-1α) plays a key role in the over-expression of glucose transporters and glycolytic enzymes [19]. After HIF-1α is decreased in cancer cells, the Warburg effect in the same cells is decreased as well [13].

**Fig. 1:**
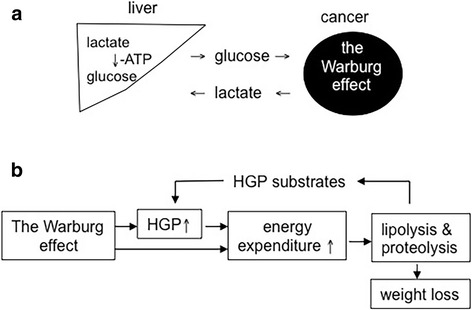
The Warburg effect and cancer cachexia **a**. The Warburg effect in cancer cells increases glucose expenditure and lactate production in the tumour carrier. The cancer-produced lactate is recycled to glucose in the liver. When the glucose is put back in the blood, cancer cells may take it for glycolysis again. **b** The Warburg effect increases both energy expenditure and hepatic glucose production (HGP). Thus, fat lipolysis and skeletal-muscle proteolysis increase to mobilize more glucose precursors. Consequently, body weight decreases

The Warburg effect in cancer cells increases total expenditure of glucose and in the meantime produces lactate as waste. In the liver, the lactate is recycled to glucose at cost of energy (Fig. 1a). When the glucose is released into the circulation, cancer cell may take it for glycolysis again (Fig. 1a). The futile glucose-lactate shuttle is called Cori cycle that increases energy expenditure and hepatic gluconeogenesis (Fig. 1b) [20]. Consequently, fat and skeletal muscle undergoes catabolic metabolisms to mobilize more glucose precursors for gluconeogenesis. When such conditions persist, body weight decreases. In this light, the Warburg effect in cancer cells hypothetically triggers cancer cachexia (Fig. 1b). In keeping with this hypothesis were the results from one of our previous studies: When wild-type human pancreatic cancer cells were transplanted in growing athymic mice, the mice showed decreased body-weight gain; when the HIF-1α gene was silenced to inhibit the Warburg effect in the cancer cells, the tumour carrier’s body weight was improved [13].

So far, it is unclear whether the Warburg effect in cancer cells induces cachexia independent of other cachexia-inducing abilities the cancer cells possess. It is also unclear whether the levels of cancer-cell glycolysis determine the levels of hepatic gluconeogenesis, fat lipolysis, and skeletal-muscle proteolysis in cancer cachexia. In the present study, we sought to address these questions. However, when cancer cells grow in vivo, they may exercise all cachexia-inducing capabilities to induce the disease, so it is difficult to single out the contribution made by the Warburg effect to the genesis of cancer cachexia.

To overcome this obstacle, we examined five human pancreatic cancer cells for their glycolysis and secretion of TNF-α, IL-1β, and IFN-γ. As a result, we selected for the present study two cell lines namely MiaPaCa2 and AsPC1. The glycolysis levels were typically high in MiaPaCa2 cells and exceptionally low in AsPC1 cells. In addition, both MiaPaCa2 and AsPC1 cells were competent in the secretion of TNF-α, IL-1β, and IFN-γ. In one experiment, these cell lines were implanted in different athymic mice, so the cancer cells may exert all capabilities to induce cachexia. In another experiment, we used the supernatants of these cell lines to treat normal mice to see whether soluble factors from these cells induced cachexia. When data from these models were compared with each other, the role of the Warburg effect in the induction of cancer cachexia was revealed.

## Methods

### Animals and cancer cells

Normal and athymic Balb/c mice (male) were bought from Hua-Fu-Kang Bioscience (Beijing, China). When mice arrived, they were 4 or 5 weeks old and weighed 17−23 g. After acclimation, they were randomly designated to experimental groups. Throughout experiment, they lived in a room with 12 h/12 h light/dark cycle and had free access to chow and water.

We bought from the Cell Bank of Chinese Academy of Science (Shanghai, China) five human pancreatic cancer cell lines, i.e. AsPC1 (#CC2404), BxPC3 (#CC2405), HPAF-2 (#CC2407), MiaPaCa2 (#CC2408), and Panc-1 (#CC2401), as well as C2C12 mouse myoblasts (#CC9003). Unless indicated otherwise, all cells were cultured at 37°C in normoxia (95% air and 5% CO_2_), using RPMI-1640 media and Dulbecco modified Eagle’s media (DMEM) supplemented with foetal bovine serum (FBS, 10%), glutamine (2 mM), penicillin (100 U/ml), and streptomycin (100 μg/ml). Culture media and supplements were bought from the distributor of Gibco Thermo Fisher Scientific in Beijing (China).

### Pancreatic cancer cells’ Warburg effect and cytokine secretion in vitro

AsPC1, BxPC3, HPAF-2, MiaPaCa2, and Panc-1 cells were cultured till 90% confluence. After rinsing with phosphate buffered saline, these cells were cultured in serum-free media for 6 h in normoxia or hypoxia (1% O_2_, 5% CO_2_, 94% N_2_) [13]. After whole-cell proteins were extracted, glucose transporter-1 (Glut1), hexokinase-2 (HK-II), and phosphofructokinase-1 (PFK-1) were determined by Western blot. Glucose, lactate, TNF-α, IL-1β, and IFN-γ were assayed in removed media. Rongsheng Life Pharmacological (Shanghai, China) and Jiancheng Bio-engineering (Nanjing, China) produced the kits for the glucose and lactate assays. Human TNF-α, IL-1β, and IFN-γ were determined, using ELISA kits from Four-A Biotech (Beijing, China).

### Transplantation of MiaPaCa2 and AsPC1 cells in athymic mice

MiaPaCa2 and AsPC1 cells were suspended in RPMI-1640 media and transplanted subcutaneously in athymic mice (3 × 10^6^ cells/mouse), giving a group of MiaPaCa2-cell carriers (*n* = 10) and a group of AsPC1-cell carriers (*n* = 13). Intact athymic mice were used as normal controls (*n* = 14). In the next 8 weeks, food intake and body weight were recorded on a weekly basis. In the end of week 8, all mice were anesthetized, using 5% chloral hydrate. Blood was collected from the orbital sinus and centrifuged (1500 x g, 10 min, 4°C) to obtain plasma. After mice were killed by cervical dislocation, subcutaneous tumour and inguinal fat pads were removed and weighed. Skeletal muscle was removed from hind legs. The abdominal cavity was opened, epididymal fat pads were removed and weighed, and the liver was sampled. Plasma and tissue samples were kept at − 80°C.

### Treating normal mice with the supernatants of MiaPaCa2 or AsPC1 cells

MiaPaCa2 and AsPC1 cells were cultured in different Petri dishes (diameter = 10 cm) till 90% confluence. Then, the cells were incubated in 15 ml serum-free RMPI-1640 medium for 24 h under normoxic conditions. The media that were conditioned by MiaPaCa2 and AsPC1 cells, respectively, were collected. The media were centrifuged to remove debris and then were saved for experiment.

Normal Balb/c mice were divided in three groups (6 mice per group). Then, they were subjected to subcutaneous injection (0.5 ml, twice a day) of normal control medium or the media that were conditioned by MiaPaCa2 and AsPC1 cells, respectively. After 7 days, all mice were sacrificed as in the preceding experiment.

In a follow-up experiment, normal Balb/c mice were divided in three groups. Mice in two groups (10 mice per group) were subjected to subcutaneous injection of normal control medium or the MiaPaCa2-cell conditioned medium as in the preceding experiment (0.5 ml, twice a day). The mice in the third group (*n* = 11) were subjected to subcutaneous injection of an increased amount of the MiaPaCa2-cell conditioned medium (1.0 ml, twice a day). After 7 days, all mice were sacrificed as described before.

### Incubating skeletal-muscle cells with media conditioned by MiaPaCa2 cells

C2C12 mouse myoblasts were cultured in 6-well plates, using DMEM containing 10% FBS. When cells were 95% confluent, they were cultured for 48 h in DMEM with 2% horse serum so as to differentiate to skeletal-muscle cells. Then, the cells were incubated for 4 h in normal control medium or in the medium that was conditioned by MiaPaCa2 cells. Intracellular atrogin-1 and myosin (heavy chain) were determined by Western blotting.

### Western blots

We performed Western blots to determine Glut1, HK-II, PFK-1, pyruvate carboxylase (PCB), glucose-6-phosphatase (G-6-Pase), LMF, PIF, atrogin-1, muscle ring finger-1 (MURF1) protein, myosin (heavy chain), insulin-like growth factor binding protein (IGFBP)-3, and adipose triglyceride lipase (ATGL). β-Actin and β-tubulin were used as loading controls. Santa Cruz Biotechnology (Santa Cruz, CA) produced the antibodies for HK-II (#6521), PFK-1 (#377346), LMF (#11238), G-6-Pase (#27196), PCB (#43228), and β-actin (#47778). Abcam (Cambridge, UK) produced the antibodies for Glut1 (#115730) PIF (#52138), MURF1 (#172479), myosin (#124205), and ATGL (#3370–1). ECM Biosciences (Versailles, KY), R&D Systems (Minneapolis, MN), and Proteintech (Chicago, IL) produced the antibodies for atrogin-1 (#AP2041), IGFBP3 (#MAB305), and β-tubulin (#66240–1), respectively.

Tissue samples were homogenized with a mechanical homogenizer, and whole-cell proteins were extracted using RIPA lysis buffer. When proteins were extracted from cultured cells, the lysis buffer was used in the first place. Protein samples were separated in polyacrylamide gel, transferred to polyvinylidene difluoride membrane, and incubated with a primary antibody at 4°C overnight. After rinsing, the membrane was incubated with a secondary antibody at room temperature for 1 h. Specific blotting was visualized, using an enhanced ECL detection kit.

### Other assays

Plasma glucose and lactate were determined, using aforementioned kits. Plasma triglycerides were determined, using a kit produced by Jiancheng Bioengineering (Nanjing, China). To determine plasma TNF-α and IL-6, we used an ELISA kit for mouse TNF-α (#E02T0008, Bluegene Biotech, Shanghai, China) and an ELISA kit for mouse IL-6 (DKW12–2060, Dakewei Biotech, Shenzhen, China). When insulin-like growth factor-1 (IGF-1) was determined in skeletal muscle, we used an ELISA kit produced by Elabscience Biotechnology (Wuhan, China). Hepatic glycogen was determined using a kit produced by Jiancheng Bioengineering (Nanjing, China).

### Statistics

Data are mean ± SEM. To evaluate difference in groups, we employed the analysis of variance followed with Bonferroni or student-Newman-Keuls post-hoc test. The computer programs of Statistical Product and Service Solutions (version 17.0) and Graph-Pad Prism (version 5.01) were used. *P* < 0.05 was considered statistically significant.

## Results

### Pancreatic cancer cell lines’ Warburg effect and cytokine secretion

**Fig. 2:**
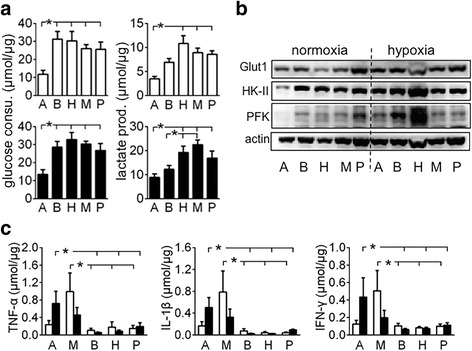
Pancreatic cancer cell’s Warburg effect and cytokine secretion AsPC1 (**a**), BxPC3 (**b**), HPAF-2 (H), MiaPaCa2 (M), and Panc-1 (P) pancreatic cancer cells were incubated for 6 h in normoxia or hypoxia. **a** Glucose and lactate were determined in removed media to assess glucose consumption and lactate production by the cells. Original data were normalized with cellular protein. White bars = normoxia, black bars = hypoxia, *n* = 12, *P < 0.05. **b** Glucose transporter-1 (Glut1), hexokinase-2 (HK-II), and phosphofructokinase-1 (PFK-1) were determined by Western blot. **c** TNF-α, IL-1β, and IFN-γ were determined in removed media. Data were normalized by cellular protein. White bars = normoxia, black bars = hypoxia, *n* = 6, * *P* < 0.05

Five pancreatic cancer cell lines were incubated for 6 h in normoxia or hypoxia. Glucose and lactate were determined in removed media, and the resulting data were used to assess the Warburg effect. In both normoxia and hypoxia, BxPC3, HPAF-2, MiaPaCa2, and Panc-1 cells had similar levels of Warburg effect, but AsPC1 cells had lower levels of Warburg effect (Fig. 2a). Generally speaking, Glut1, HK-II, and PFK-1 expression were less in AsPC1 cells, than in the other cell lines (Fig. 2b). Further, MiaPaCa2 and AsPC1 cells secreted more TNF-α, IFN-γ, and IL-1β, than the remaining cell lines (Fig. 2c).

### Energy homeostasis in athymic mice carrying MiaPaCa2 or AsPC1 cells

**Fig. 3:**
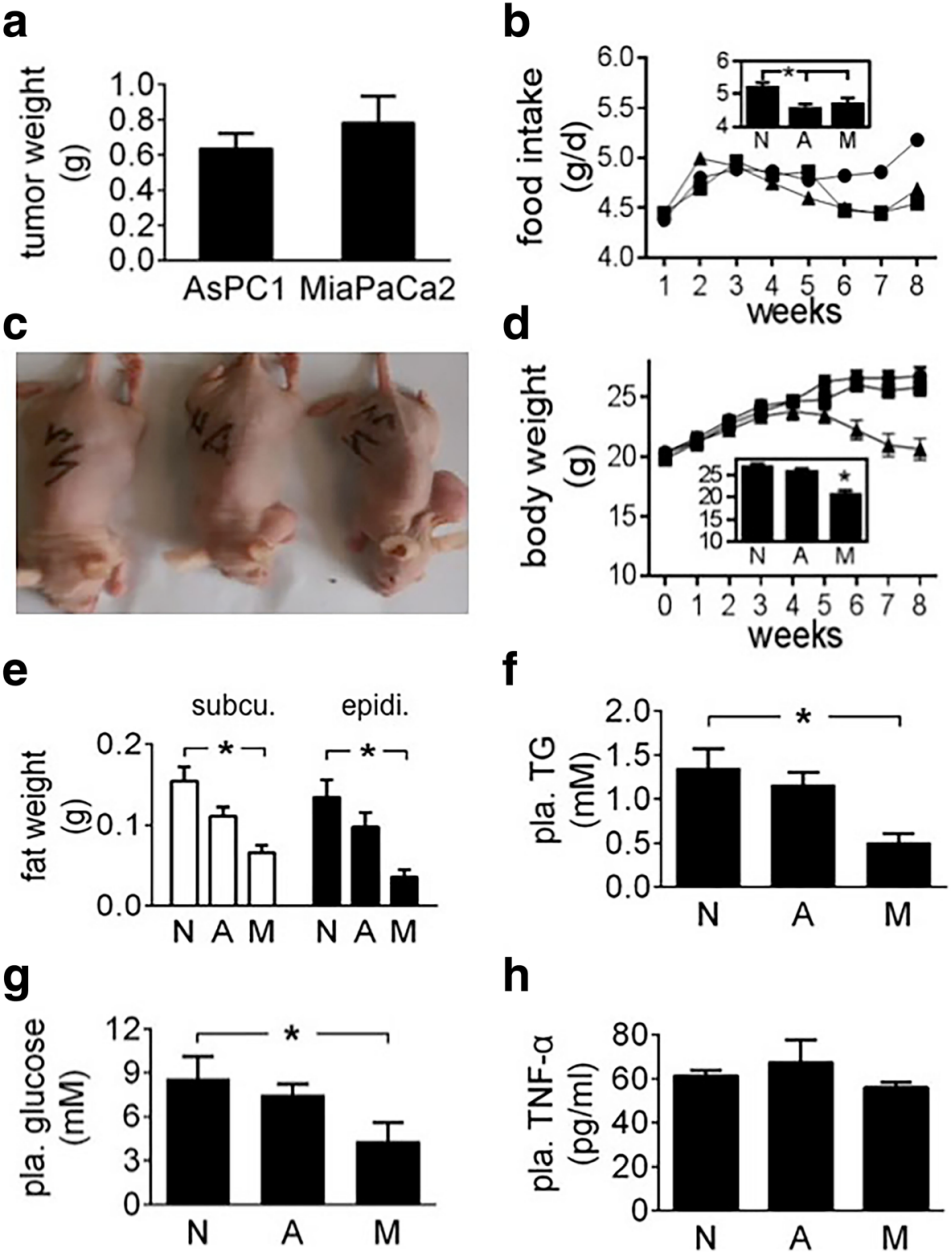
Energy homeostasis in athymic mice carrying pancreatic cancer cells MiaPaCa2 and AsPC1 cells were transplanted subcutaneously in different athymic mice for 8 weeks, giving group M (*n* = 10) and group A (*n* = 13), respectively. Normal athymic mice were used for control (group N, *n* = 14). **a** Tumour weight. **b** Weekly food intake was plotted. The data of 8 individual weeks were averaged and the results are shown in the inset. **c** Nutritional states in 3 mice representative of groups N (left), A (central), and M (right), respectively. **d** Body weight was plotted for the 8 weeks. The final body weight is shown in the inset. **e** The weight of subcutaneous and epididymal fat. **f**-**h** Plasma triglyceride (TG), glucose, and TNF-α levels. Data are mean ± SEM (See *n* in the parentheses). *P < 0.05

The subcutaneous tumours made of MiaPaCa2 and AsPC1 cells had similar weight (Fig. 3a). Both groups of tumour carriers had anorexia, compared to intact mice (Fig. 3b). The body weight of the mice that carried MiaPaCa2 cells was decreased, as compared to the control value (Fig. 3c and d). No significant decrease was seen in the body weight of the mice that carried AsPC1 cells.

The weight of inguinal and epididymal fat was decreased significantly in the carriers of MiaPaCa2 cells but not AsPC1 cells, as compared to normal values (Fig. 3e), which suggests that lipolysis increased in the former group of tumour carriers but not the latter. Plasma triglycerides were decreased in the carriers of MiaPaCa2 cells, compared to normal value (Fig. 3f), which may a result of increased triglyceride consumption by the tumour carriers [21, 22]. Plasma glucose was decreased in the mice carrying MiaPaCa2 cells, compared to normal value (Fig. 3g). This result was essentially identical to that demonstrated before [23]. Plasma triglyceride and glucose levels were normal in the carriers of AsPC1 cells (Fig. 3f and g). Plasma levels of lactate were normal in both groups of tumour carriers, compared to the control value in the intact mice (data not shown).

Cachectic cytokines in cancer patients are derived from both neoplastic and non-neoplastic cells [1, 2, 5]. In the athymic mouse experiment, we determined plasma level of mouse TNF-α and used it as an index of cachectic cytokines. TNF-α levels in two groups of tumour carriers were not significantly different from those seen in the intact mice (Fig. 3h). This result may be due to the fact that the immune system in athymic mice is incompetent, so the mice in the present study did not release TNF-α in response to the cancer cells. PIF and LMF are cancer-induced cachectic factors [6–8]. We used Western blot to determine PIF and LMF in the plasma of athymic mice. As a result, we found neither of them therein, no matter the mice carried tumours or not (data not shown).

**Fig. 4:**
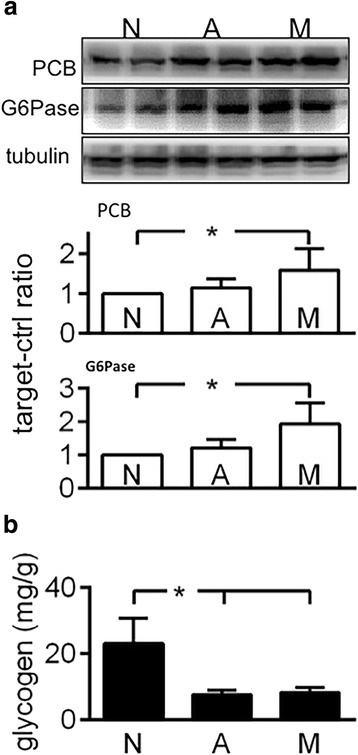
The effect of tumour carriage on liver metabolism Liver tissues were obtained from normal athymic mice (group N, *n* = 14) and from those that carried MiaPaCa2 (group M, *n* = 10) or AsPC1 cells (group A, *n* = 13). **a** Western blots were performed to determine pyruvate carboxylase (PCB) and glucose-6-phosphatase (G6Pase), using β-tubulin as a loading control. The blots are representative data. The histograms show the results of all mice. **b** Glycogen was determined. Data are mean ± SEM (See *n* in the parentheses). *P < 0.05

**Fig. 5:**
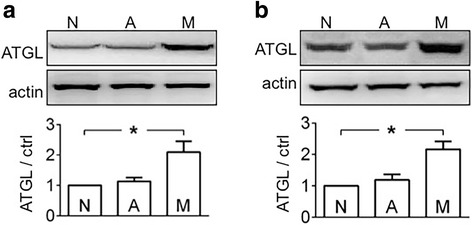
The effect of tumour carriage on fat lipolysis Inguinal (**a**) and epididymal (**b**) fat were obtained from normal athymic mice (group N, *n* = 14) and from those that carried MiaPaCa2 (group M, *n* = 10) or AsPC1 cells (group A, *n* = 13). Western blots were performed to determine adipose triglyceride lipase (ATGL), using β-actin as a loading control. The blots are representative data. The histograms show the results of all mice (See *n* in the parentheses). *P < 0.05

To assess the effect of tumour carriage on hepatic gluconeogenesis, we checked PCB and G-6-Pase expression in the liver. PCB and G-6-Pase expression were increased significantly when athymic mice carried MiaPaCa2 cells, as compared to reference values seen in intact mice (Fig. 4a). This result suggests that hepatic gluconeogenesis was increased in the mice carrying MiaPaCa2 cells. No significant changes in PCB and G-6-Pase expression were seen when athymic mice carried AsPC1 cells (Fig. 4a). However, hepatic glycogen was decreased significantly in both groups of tumour carriers (Fig. 4b), compared to reference value in intact mice. ATGL regulates cancer-induced lipolysis [24]. When athymic mice carried MiaPaCa2 cells, ATGL expression was increased in both inguinal and epididymal fat pads, compared to reference values in intact mice (Fig. 5). This result suggests that adipose tissues in these tumour carriers underwent increased lipolysis. No significant increase was seen in ATGL expression when athymic mice carried AsPC1 cells (Fig. 5).

**Fig. 6:**
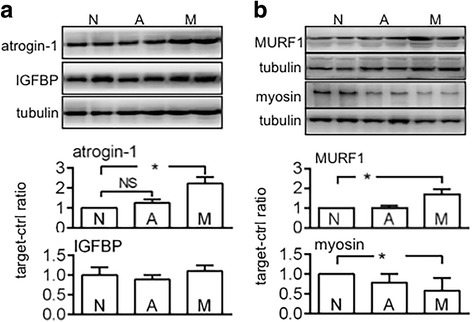
The effect of tumour carriage on skeletal muscle Skeletal-muscle samples were obtained from normal athymic mice (group N, *n* = 14) and from those that carried MiaPaCa2 (group M, *n* = 10) or AsPC1 cells (group A, *n* = 13). Western blots were performed to determine atrogin-1 (**a**), IGFBP-3 (**a**), myosin (**b**), and MURF1 (**b**), using β-tubulin as a loading control. The blots are representative data. The histograms show the results of all mice (See *n* in the parentheses). *P < 0.05, NS: not significant

**Fig. 7:**
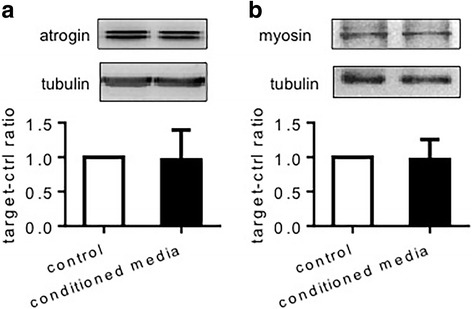
Atrogin-1 and myosin expression by C2C12 cells in vitro C2C12 cells were incubated for 4 h in normal control medium or MiaPaCa2 cell-conditioned medium. Atrogin-1 and myosin were determined by Western blot, using β-tubulin as a loading control. The blots are representative data. The histograms summarize data from 6 experiments

Skeletal-muscle proteolysis is regulated by atrogin-1 and MURF1, and skeletal-muscle protein biosynthesis is regulated by IGF-1 [25–27]. In addition, the amount of free (active) IGF-1 is regulated by IGFBPs [28, 29]. In the present study, the athymic mice carrying MiaPaCa2 cells showed increased atrogin-1 and MURF1 expression, normal IGFBP-3 expression, and decreased myosin expression in skeletal muscle, compared to reference values in intact mice (Fig. 6). However, IGF-1 contents in the same skeletal-muscle samples were similar to the normal value in intact mice (data not shown). Thus, the skeletal muscle in the carriers of MiaPaCa2 cells had an increased proteolysis without compensation in protein biosynthesis. When the same parameters were checked in the athymic mice that carried AsPC1 cells, no significant changes were seen (Figs. 6a-c). When we incubated C2C12 skeletal-muscle cells in the medium conditioned by MiaPaCa2 cells, the cells showed normal atrogin-1 and myosin expression, compared to reference data seen in the C2C12 cells that were incubated in normal medium (Fig. 7). Thus, the supernatants of MiaPaCa2 cells may not induce proteolysis in skeletal-muscle cells.

### Energy homeostasis in mice treated with the supernatants of MiaPaCa2 or AsPC1 cells

**Fig. 8:**
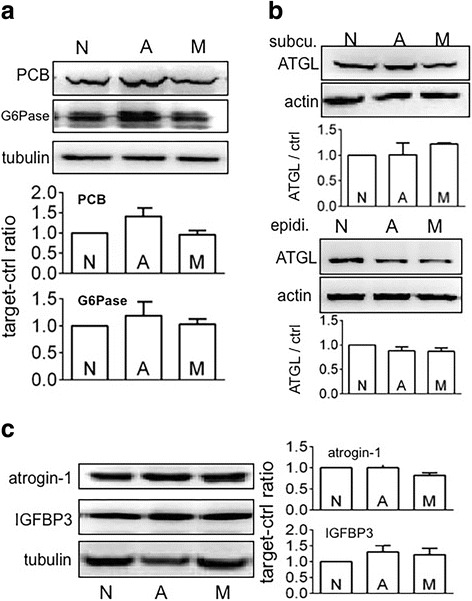
The effects of MiaPaCa2 or AsPC1-cell supernatants on hepatic, fat, and skeletal-muscle metabolisms Normal mice in 3 groups (6 mice/group) were subjected to subcutaneous injection (0.5 ml, twice a day) of normal control medium (group N) or the media that were conditioned by MiaPaCa2 cells (group M) or by AsPC1 cells (group A). After 7 days, all mice were sacrificed. Their liver, fat, and skeletal muscle were obtained. Western blots were performed, using β-tubulin and β-actin as loading controls. **a** PCB and G6Pase expression in the liver. **b** ATGL expression in subcutaneous and epididymal fat. **c** Atrogin-1 and IGFBP-3 expression in skeletal muscle. Blots are representative results. The histograms show the results of all mice

When athymic mice carried MiaPaCa2 cells, the expression of PCB, G-6-Pase, ATGL, atrogin-1, MURF1, and myosin were changed in the liver, fat, and skeletal muscle, respectively. If these changes were induced by humoral factors that were released by MiaPaCa2 cells, the same results may be seen again when normal mice were subjected to the supernatants of MiaPaCa2 cells. After we treated normal mice with the supernatants of MiaPaCa2 and AsPC1 cells, we did not see any significant changes in the expression of PCB, G-6-Pase, ATGL, atrogin-1, and IGFBP-3, as compared to reference values seen in the mice that were treated with vehicle (Fig. 8).

**Fig. 9:**
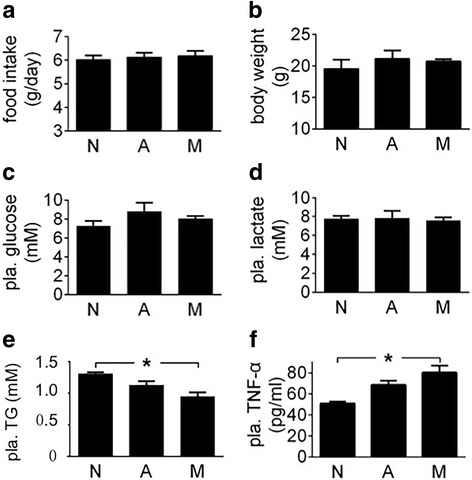
The effects of MiaPaCa2 or AsPC1-cell supernatants on energy homeostasis See the legend of Fig. 8 for study design. **a** Daily food intake was averaged for the 7-day experiment. **b** Final body weight. **c**-**f** Plasma levels of glucose, lactate, triglycerides (TG), and TNF-α; *P < 0.05

**Fig. 10:**
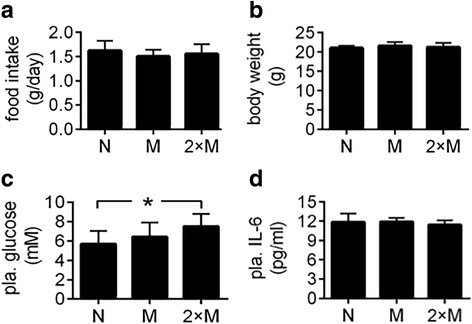
The effects of different amounts of MiaPaCa2-cell supernatants on energy homeostasis Mice in 2 groups (10 mice/group) were injected (s.c.) with 0.5 ml of MiaPaCa2-cell supernatants (group M) or vehicle (group N) for 7 days. In the meantime, mice in a third group (*n* = 11) were injected with 1.0 ml of the MiaPaCa2-cell supernatants (group 2×M). **a** Daily food intake was averaged for the 7 days. **b** Final body weight. **c** Plasma levels of glucose. **d** Plasma levels of IL-6. *P < 0.05

After normal mice were treated with MiaPaCa2- or AsPC1-cell supernatants, food intake, body weight, and plasma levels of glucose and lactate were not changed significantly, as compared to reference values seen in the mice treated with vehicle (Fig. 9a−d). Plasma triglycerides were decreased when mice were treated with the supernatants of MiaPaCa2 cells but not AsPC1 cells, compared to reference value seen in the mice treated with vehicle (Fig. 9e). Of note, the decrease in plasma triglycerides was comparable to that seen when athymic mice carried MiaPaCa2 cells (Fig. 3f). Taken together, MiaPaCa2 cells may secrete something that increased the utilization of triglycerides in these mice. When mouse TNF-α was determined in plasma, a significant increase was seen in the mice that were treated with the supernatants of MiaPaCa2 cells but not AsPC1 cells, as compared to reference value seen in the mice treated with vehicle (Fig. 9f). In the follow-up experiment, we treated normal mice with two doses of MiaPaCa2-cell supernatants, one being as in the preceding experiment and the other being twice as much. The increase in MiaPaCa2-cell supernatants did not change food intake and body weight, but it did induce a significant increase in plasma glucose (Fig. 10a-c). IL-6 may be a key regulator of cancer cachexia [30]. However, MiaPaCa2 cells did not release IL-6 [31]. When we determined mouse IL-6 in the plasma, no significant difference was found in the different groups of mice (Fig. 10d).

## Discussion

In recent years, a big progress has been made in the research for cancer cachexia [32]. For instance, there is an international consensus on both definition and classification of cancer cachexia [33]. In addition, ketone-body metabolism is known to play a role in cancer cachexia [34]. The mechanism by which cancer cachexia suppresses anti-tumour immunity is defined [30, 35]. So is the mechanism by which malignant tumours trigger white adipose tissue browning [36–38].

Several lines of evidence have supported the hypothesis that the Warburg effect in cancer cells triggers cancer cachexia [13–17]. However, this hypothesis has not been tested systemically. In the present study, high-glycolytic MiaPaCa2 cells increased hepatic gluconeogenesis, fat lipolysis, and skeletal-muscle proteolysis in the athymic mice carrying the cancer cells. On the other hand, energy homeostasis in the athymic mice carrying low-glycolytic AsPC1 cells was largely normal with the exception of anorexia and decreased hepatic glycogen. Anorexia is usually induced by anorectic cytokines (e.g. TNF-α, IFN-γ, and IL-1β) and neuropeptides [1, 39]. When these factors are increased in cancer patients, they attack the part of hypothalamus that regulates appetite to induce anorexia [1].

Numerous studies have shown evidence that glucose, lipid, and protein turnover are increased in cancer patients [14–17]. Radioactive tracers are usually used to demonstrate the increase in nutrient turnover [14–16]. Sometimes, cancer-induced increase in glucose turnover is seen as a decrease in circulating glucose [23]. In keeping with this notion, plasma glucose was decreased when athymic mice carried high-glycolytic MiaPaCa2 cells.

Cancer cells may induce cachexia by secreting cachectic cytokines [1, 2]. In addition to cancer cells, macrophages and other non-cancer cells may release cachectic cytokines in the presence of cancer [1, 5]. When cachectic cytokines are increased in peripheral circulation, they may target liver, skeletal muscle, and fat to induce cachexia. In the present study, both MiaPaCa2 and AsPC1 cells secreted cachectic TNF-α, IL-1β, and IFN-γ in vitro. However, energy homeostasis was largely undisturbed when normal mice were treated with the supernatants of MiaPaCa2 and AsPC1 cells. Thus, the cachexia seen in the athymic mice carrying MiaPaCa2 cells may not be induced by humoral factors released by these cells.

In the present study, MiaPaCa2 and AsPC1 cells did not release PIF and LMF. Using immunohistochemical methods, Kamoshida and co-workers looked for PIF and LMF in five human pancreatic cancer cell lines (including MiaPaCa2) carried by athymic mice. Three cell lines (including MiaPaCa2) had neither PIF nor LMF, and two cell lines showed weak expression of PIF or LMF, respectively [9].

Orthotopic and subcutaneous transplantation of human pancreatic cancer cells in athymic mice are two models that are frequently used in pancreatic-cancer research. When pancreatic cancer cells are transplanted orthotopically, they may cause ascetic fluid, jaundice, and liver metastasis, and these intra-abdominal complications may induce cachectic states directly [40]. This being the case, we chose the subcutaneous model for the present study. Unfortunately, the subcutaneous model cannot be used to study how the endocrine pancreas is involved in pancreatic cancer-induced cachexia. However, previous studies have showed that the endocrine pancreas is impaired in pancreatic cancer, and the impairment in turn contributes to the pathogenesis of pancreatic cancer-induced cachexia [3, 4, 10–12, 41, 42]. For instance, when pancreatic cancer was induced in hamsters, the endocrine pancreas showed a decrease in insulin-producing cells and an increase in other hormonal cells [41]. In addition, the circulating profiles of pancreatic hormones were changed in the hamsters with pancreatic cancer [42]. Similar abnormalities in the anatomy and function of the endocrine pancreas are also seen in pancreatic cancer patients [3, 4, 10–12].

Data from the present study suggest that the Warburg effect in pancreatic cancer cells drives the pathogenesis of pancreatic cancer-induced cachexia. Inhibiting the Warburg effect in pancreatic cancer cells may attenuate the cachexia induced by pancreatic cancer [13, 43, 44].

## Conclusion

The Warburg effect in pancreatic cancer cells triggers metabolic abnormalities in liver, fat, and skeletal muscle and thus induces cachexia. Inhibiting the Warburg effect in pancreatic cancer cells may help the tumour carrier restore energy homeostasis.

## Abbreviations

ATGL: Adipose triglyceride lipase
ELISA: Enzyme-linked immunosorbent assay
G-6-Pase: Glucose-6-phosphatase
Glut1: Glucose transporter-1
HGP: Hepatic glucose production
HK-II: Hexokinase-2
IFN-γ: Interferon-γ
IGF-1: Insulin-like growth factor-1
IGFBP: IGF binding protein
IL: Interleukin
LMF: Lipid mobilizing factor
MURF1: Muscle ring finger-1
PCB: Pyruvate carboxylase
PFK-1: Phosphofructokinase-1
PIF: Proteolysis inducing factor
TNF-α: Tumour necrosis factor-α
